# Engineering a High-Fidelity Neonatal Silicone Phantom: Development, Optimization, and User Evaluation of a 3D-Printed Vascular Access Model

**DOI:** 10.7759/cureus.102315

**Published:** 2026-01-26

**Authors:** Shaylin D Zoellner, Aneesha Morris, Aarnav Parikh, Pratik Parikh

**Affiliations:** 1 Pediatrics, University of the Incarnate Word School of Osteopathic Medicine, San Antonio, USA; 2 Pediatrics, Baylor College of Medicine, San Antonio, USA; 3 Biology, BASIS San Antonio, Shavano Campus, San Antonio, USA

**Keywords:** 3d printing, accessible medical simulation device, pediatrics and neonatology, skills and simulation training, ultrasound phantom

## Abstract

Securing intravenous (IV) access in neonates is technically challenging due to extremely small vessel caliber, fragile skin, and limited subcutaneous tissue. High-fidelity training phantoms are essential for teaching ultrasound-guided vascular access, yet most commercial models are adult-sized, lack neonatal realism, and are cost-prohibitive.

The aim of this study was to design and optimize a low-cost, ultrasound-compatible 3D silicone phantom that replicates neonatal tissue and vasculature for peripheral and central IV access training.

A multi-phase design process incorporated 3D-printed polylactic acid (PLA) molds, Dragon Skin™ silicone (Smooth-On, Inc., Macungie, PA, USA), Slacker® softener (Smooth-On, Inc., Macungie, PA, USA), and 3% talcum powder to enhance echogenicity. Silicone tubing (0.2-0.4 mm internal diameters (ID)) was embedded at neonatal-appropriate depths using rotational casting for dermal uniformity and vessel channel supports for positional accuracy.

Iterative engineering improved dermal wall uniformity, vessel stability, and ultrasound visibility, resulting in a durable, reproducible phantom with realistic tissue compliance and vessel compressibility.

The developed 3D silicone phantom provides a realistic, affordable, and reproducible neonatal vascular access simulator. User feedback supports its fidelity and utility, and its low material cost enables broad implementation in neonatal procedural training programs.

## Introduction

Intravenous (IV) access is one of the most frequently performed but technically demanding procedures in neonatal intensive care units (NICUs). Neonates, particularly those born preterm or with low birth weight, often require vascular access for the administration of fluids, medications, parenteral nutrition, and blood products. However, their small vasculature, thin skin, and limited subcutaneous tissue pose significant challenges to successful cannulation [1,2]. These anatomical constraints not only increase the risk of failed attempts and procedural trauma but can also delay life-saving interventions. Therefore, ensuring that neonatal providers develop high levels of skill and confidence in vascular access is essential for both procedural safety and clinical outcomes.

Ultrasound guidance has emerged as a transformative adjunct in vascular access, enabling the direct visualization of vessels and needle advancement. Studies have consistently shown that ultrasound-guided peripheral IV access improves cannulation success, reduces the number of attempts, minimizes complications, and enhances patient safety across all pediatric age groups [3]. Despite its growing use in neonatology, ultrasound-guided IV placement remains a high-skill technique that is heavily reliant on operator training and experience. For novice learners and even seasoned clinicians, ongoing hands-on practice is critical to mastering this modality.

Simulation-based training offers a risk-free and reproducible environment to acquire and refine procedural skills [4]. The use of phantoms, a synthetic model used to simulate human tissue for medical training, or task trainers for ultrasound-guided access have gained popularity across pediatric and adult settings [5]. However, a critical gap exists in the availability of anatomically and physiologically appropriate models for neonates. Most commercial phantoms are designed for adult or general pediatric use, lacking the necessary fidelity or accuracy to simulate the unique characteristics of neonatal tissue, vessel size, and depth [5]. Furthermore, high-fidelity models that approximate neonatal anatomy are often prohibitively expensive and inaccessible to many training programs [6]. As a result, neonatology trainees may be forced to practice on live patients, which raises ethical concerns and exposes fragile neonates to unnecessary risk.

This study aimed to design and develop a novel 3D silicone phantom for neonatal ultrasound-guided IV placement. Our objective was to create a cost-effective, customizable, and anatomically accurate training phantom that simulates both peripheral and central vessel cannulation. Leveraging affordable materials, desktop 3D printing, and an iterative prototyping approach, we sought to optimize tissue mimicry, vessel echogenicity, and ultrasound compatibility. In doing so, we also aimed to democratize access to high-fidelity simulation tools and lay the groundwork for broader implementation in neonatal education.

## Technical report

The development of a high-fidelity, ultrasound-compatible neonatal silicone phantom followed a structured four-phase approach: (1) selection of materials, (2) design and fabrication of the 3D-printed mold, (3) construction of the multilayered silicone phantom, and (4) final assembly and optimization.

Selection of materials

The choice of phantom materials was based on three main criteria: physical resemblance to skin and subcutaneous tissue, compatibility with ultrasound imaging, and durability for repeated use. Prior literature describes phantoms made from water-based hydrogels, rubbers, and silicone polymers, each with distinct advantages and limitations [7].

Based on these considerations, Dragon Skin™ silicone (Smooth-On, Inc., Macungie, PA, USA) was selected for its skin-like consistency, tensile strength, biocompatibility, and tolerance to a wide temperature range (−53°C to +204°C). Several variants (Dragon Skin 10 Fast, 20, and FX-Pro) were evaluated.

To achieve realistic pliability mimicking neonatal subcutaneous tissue, Slacker® silicone softener (Smooth-On, Inc., Macungie, PA, USA) was added in varying ratios. Slacker modifies the silicone's viscosity and cure properties, increasing its elasticity and creating a more lifelike feel with a self-sealing surface suitable for repeated needle insertion.

To enhance ultrasound echogenicity, talcum powder was selected based on its proven use in simulation literature, low cost, and ease of incorporation. Other echogenic agents, such as polyacrylamide or oil-based additives, were not chosen due to mixing difficulty and variability. Preliminary experiments tested talc concentrations between 3% and 5% by volume, with 3% producing the optimal balance between echogenic enhancement and avoidance of posterior acoustic shadowing.

Silicone tubing (0.2 mm and 0.4 mm internal diameters (ID)) was used to represent peripheral and central neonatal vessels. Tubes were selected for their flexibility, compressibility, and ultrasound visibility and were positioned to reflect realistic anatomical depth and orientation.

Design and fabrication of the 3D-printed mold

The 3D-printed mold was a critical component in the creation of the neonatal silicone phantom, allowing for the consistent shaping of the external dermis layer and precise placement of simulated vasculature. The design process underwent multiple iterations to accommodate anatomical realism, structural integrity, ease of use, and repeatability.

Initial Design and Prototyping

Mold prototypes were designed using Tinkercad (Autodesk, San Francisco, CA, USA), a browser-based computer-aided design (CAD) software. These designs were exported in stereolithography (STL) format and printed using a Tronxy XY-2 Pro 3D printer with polylactic acid (PLA) filament and a 0.2 mm nozzle diameter, chosen to ensure high-resolution edge fidelity and fine anatomical detail. The first-generation mold featured a rectangular base with a fixed wall height, vertical sidewalls with open slots through which silicone tubing could be threaded, and a single open cavity to hold the tissue-mimicking silicone.

However, this configuration resulted in inconsistent tubing placement. The open side slots did not support the tubing during silicone pouring, allowing it to sag under its own weight or float within the uncured silicone, leading to improper vessel depth and alignment. Additionally, removal of the mold post-curing sometimes damaged the thin dermal walls or dislodged tubing.

Design Revisions

To resolve these issues, a second-generation mold was developed with the following features: First is detachable side panels, that is, two individual side walls that could be removed post-curing without stressing the phantom. Second is embedded horizontal cylindrical channels, which were added to allow the secure threading of silicone tubing through pre-defined entry and exit ports. Tubing could be inserted before or during the curing process, maintaining fixed orientation and depth. Third is rounded internal corners, which minimized silicone pooling in sharp edges and reduced air bubble entrapment. Fourth is curved base geometry, which enhanced the ability to perform rotational coating of the silicone against the walls during the dermis fabrication step. Fifth and last is central cavity volume, which was sized to hold approximately 100 mL of total silicone volume, sufficient for dermal and inner matrix layers.

**Figure 1:**
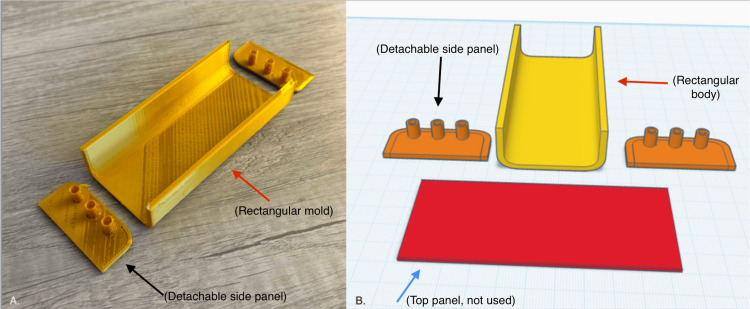
Final PLA filament 3D-printed mold with rounded side walls and corresponding CAD design file Panel A (left image): photograph of the final iteration 3D-printed mold fabricated using PLA filament on a Tronxy XY-2 Pro 3D printer with 0.2 mm nozzle diameter. Scale reference: mold base dimensions accommodate neonatal forearm or scalp vein pattern simulation. Panel B (right image): CAD rendering created using the Tinkercad (Autodesk) browser-based software, showing the modular mold assembly in exploded view. The image depicts (1) the central rectangular mold body with rounded internal corners (red arrows), (2) two detachable side panels (black arrows) featuring integrated cylindrical channel ports for vessel tubing insertion, and (3) a prototyped top panel, not utilized in the final fabrication protocol (blue arrow). The CAD design was exported in STL format for 3D printing. The cylindrical channels accommodate three vessels, one central vessel using 0.4 mm ID tubing and two peripheral vessels using 0.2 mm ID tubing, positioned to replicate anatomically accurate neonatal vasculature patterns. The modular design enables reproducible phantom construction with minimal inter-unit variation. PLA: polylactic acid; CAD: computer-aided design; ID: internal diameter; STL: stereolithography

The mold's external channels allowed for the insertion of three vessels, one central vessel (0.4 mm ID tubing) and two peripheral vessels (0.2 mm ID tubing), placed to mimic neonatal forearm or scalp vein patterns (Figure 1). 

Surface Preparation and Use

Each PLA mold was cleaned and allowed to air-dry before use. This step removed debris and minimized surface tension variations that could alter silicone curing or introduce bubbles. No mold-release agent was required, as the cured silicone detached cleanly from PLA due to their material incompatibility.

Mold Assembly for Use

During each phantom fabrication session, the mold was pre-assembled with detachable walls and secured with binder clips or elastic bands for stability. After the dermal silicone layer was poured and rotationally spread, the horizontal tubing was threaded into place during the semi-cured phase (~three to four minutes post-pour). Once the dermal and tubing layers were partially set, the inner core silicone mixture was poured in a single step to complete the phantom.

The modular mold design not only improved tubing placement accuracy and reduced air bubbles but also increased production efficiency and allowed for the repeatable construction of multiple phantoms with minimal variation.

Silicone phantom construction

The silicone phantom was developed in two primary anatomical layers: an outer dermal shell and an inner core representing subcutaneous fat and muscle. A key goal was to produce a phantom that realistically mimics neonatal soft tissue consistency, allows for superficial vessel embedding, and is compatible with high-resolution ultrasound imaging.

Dermal Layer Formation

The outer dermis layer was designed to be thin, flexible, but supportive, simulating neonatal skin with sufficient strength to withstand handling and repeated needle puncture. The dermal layer also served as the anchoring structure for synthetic vessels.

Material composition: Dragon Skin™ silicone was mixed in a 1:1 ratio (part A to part B). Two drops of thickening agent were added to increase viscosity and reduce pooling during wall application. No Slacker® was used in this layer to maintain firmness and structural support.

Wall Formation Process

Three techniques were trialed to develop an optimal dermal shell:

Manual spreading: Silicone was poured into the mold and manually brushed onto each wall. This approach resulted in irregular wall thickness, weak adhesion between corners, and poor reproducibility.

Laminated spacer technique:* *Laminated cards were cut to match the mold wall dimensions and held 0.2 mm from the wall using spacers. Silicone was injected between the wall and the card using a syringe. This method improved thickness uniformity and reduced air bubbles but required two operators and led to microcracks along the wall-base junctions upon spacer removal.

Rotational casting (final technique): Silicone was poured into the base of the mold and manually rotated side-to-side for two to three minutes, allowing the material to flow evenly along all interior surfaces. Once the silicone began to cure, excess material was drained. This method yielded smooth, consistent wall thickness, minimized air entrapment, and enhanced overall durability.

Resulting features: These were as follows: average dermal thickness: ~3 to 4 mm; surface texture: smooth, flexible, self-supporting; and adhesion to embedded vessels: excellent with no slippage or detachment.

Vessel Embedding

Simulated vasculature was constructed using flexible silicone tubing with an ID of 0.2 mm for superficial peripheral veins and 0.4 mm for deeper or central vein simulation.

The tubing was threaded horizontally through guide cylinders integrated into the mold's detachable sidewalls. To ensure optimal placement, tubing was inserted approximately three to four minutes after dermal silicone pouring when the material was semi-cured but still tacky.

Key outcomes: Vessels were embedded at a consistent depth of 3-4 mm, anatomically representative of neonatal vasculature. Partial curing allowed the vessels to bond with the dermal silicone, simulating the coupling of skin and vessels during ultrasound-guided compression or needling. The external tubing ends remained accessible, allowing for fluid infusion or the visual confirmation of needle placement.

Central Tissue-Mimicking Core

The central matrix of the phantom was designed to simulate neonatal subcutaneous tissue and muscle. It needed to be soft, elastic, and echogenic while retaining structural integrity for multiple needle insertions. Initial formulation involved just the use of Dragon Skin™ solutions A and B, which were very rigid and did not resemble neonatal central adipose tissue. The addition of Slacker® improved elasticity and recoil which mimicked neonatal tissue. Various ratios of silicone polymer and Slacker® were tested to come up with the final phantom.

**Figure 2:**
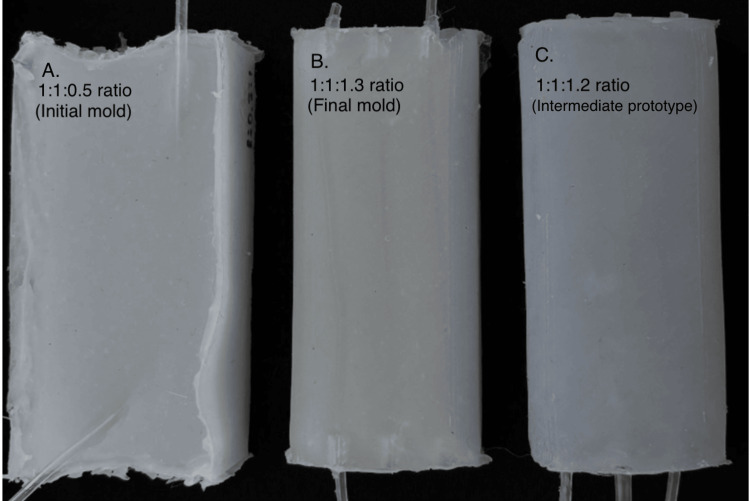
Completed silicone phantoms demonstrating the iterative formulation optimization of Dragon Skin™ and Slacker® ratios Panel A (left): initial prototype phantom constructed with Dragon Skin™ silicone in a 1:1:0.5 ratio (Dragon Skin™ part A to Dragon Skin™ part B to Slacker®). This formulation resulted in a phantom that was excessively rigid. The opacity and structural rigidity of this formulation precluded the realistic simulation of neonatal subcutaneous tissue properties. Panel B (center): final optimized phantom utilizing the definitive formulation ratio of 1:1:1.3 ratio (Dragon Skin™ part A to Dragon Skin™ part B to Slacker®) with 3% talcum powder by volume for echogenicity enhancement. This iteration most accurately replicated neonatal tissue characteristics. The translucent appearance permits the visual confirmation of embedded vasculature. Panel C (right): intermediate prototype phantom constructed with a 1:1:1.2 ratio (Dragon Skin™ part A to Dragon Skin™ part B to Slacker®). This formulation exceeded optimal softness parameters. The comparison of these three formulations illustrates the critical importance of precise Slacker® titration in achieving tissue-mimicking properties suitable for neonatal simulation. Dragon Skin™: platinum-cure silicone rubber; Slacker®: silicone softener additive

Multiple formulations of Dragon Skin™ and Slacker® were tested (Table 1, Figure 2).

**Table 1:** Iterative formulation testing of Dragon Skin™ silicone and Slacker® softener ratios: material properties and outcomes Summary of iterative formulation testing during the development of the neonatal silicone phantom. Dragon Skin™ platinum-cure silicone was mixed in equal parts (part A to part B = 1:1), with varying quantities of Slacker® silicone softener to achieve optimal tissue-mimicking properties. Five formulation ratios were systematically evaluated for (1) tissue compliance and pliability, (2) needle insertion force and resistance, (3) tissue rebound characteristics, (4) structural integrity for vessel support, and (5) material durability for repeated use. The final formulation (1:1:1.3) provided the optimal balance of softness mimicking neonatal subcutaneous tissue while maintaining adequate structural support for stable vessel positioning. This ratio was combined with 3% talcum powder by volume to achieve ultrasound echogenicity without posterior acoustic shadowing. A: Dragon Skin™ part A; B: Dragon Skin™ part B; Slacker: Slacker® silicone softener

Ratio (A:B:Slacker)	Results and material characteristics
1:1:0.5	Too rigid; high insertion force required for needle penetration; unrealistic tissue rebound not representative of neonatal soft tissue; poor tactile fidelity
1:1:0.75	Stiff consistency; mimicked adult tissue characteristics rather than neonatal; poor compliance; suboptimal for intended training application
1:1:1	Closer to target properties; improved softness; still slightly firm compared to neonatal tissue; acceptable needle resistance; approaching optimal compliance
1:1:1.2	Exceeded optimal softness; too soft and overly deformable; vessel shifting noted during handling; insufficient structural support for stable vessel positioning; reduced durability
1:1:1.3 (final)	Ideal balance achieved; optimal softness, pliability, and structural support; realistic neonatal subcutaneous tissue compliance; appropriate needle insertion resistance; self-sealing properties maintained; stable vessel positioning at 3-4 mm depth

**Figure 3:**
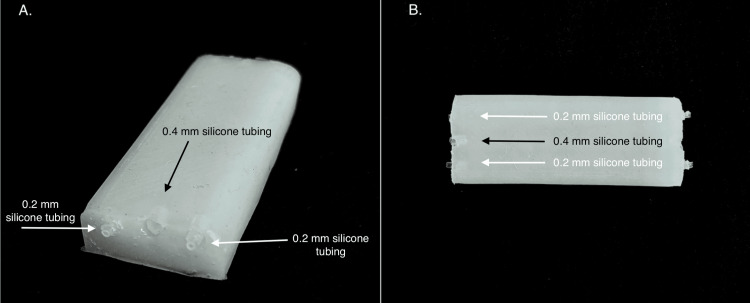
Final optimized silicone phantom demonstrating embedded vasculature configuration Panel A (left image, oblique view): oblique perspective of the completed neonatal vascular access phantom constructed using the optimized formulation (Dragon Skin™ part A to Dragon Skin™ part B to Slacker® = 1:1:1.3 with 3% talcum powder). The image demonstrates the smooth, homogeneous dermal layer surface with consistent wall thickness of approximately 3-4 mm achieved through rotational casting technique, visible embedded silicone tubing representing simulated vasculature positioned at neonatal-appropriate depths, and external tubing ends extending beyond the phantom boundaries to allow for fluid infusion during training or visual confirmation of successful cannulation. Panel B (right image, aerial view): superior aerial view of the final phantom iteration providing the visualization of the complete vessel arrangement. This perspective reveals the parallel orientation of three embedded vessels, including one central vessel (0.4 mm ID tubing simulating a larger caliber vessel, indicated by black arrows) and two peripheral vessels (0.2 mm ID tubing simulating superficial peripheral veins, indicated by white arrows), and uniform vessel spacing across the phantom surface to enable multiple cannulation attempts at varying locations. Technical specifications: dermal layer thickness, 3-4 mm; vessel depth, 3-4 mm; central vessel ID, 0.4 mm; peripheral vessel ID, 0.2 mm; total silicone volume, ~100 mL; curing time, 45-60 minutes at room temperature

The final mixture (part A to part B to Slacker = 1:1:1.3) provided a soft, gelatinous consistency closely approximating the feel of neonatal subcutaneous tissue (Figure 3). 

Echogenicity enhancement and ultrasound specifications:* *To allow ultrasound imaging of needle and vessel interactions, talcum powder was added to the silicone mixture [8]. An initial concentration of 5% total volume created excessive shadowing and obscured vessel visualization; however, the final concentration of 3% provided sufficient echogenicity for structural visualization without posterior artifact. 

**Figure 4:**
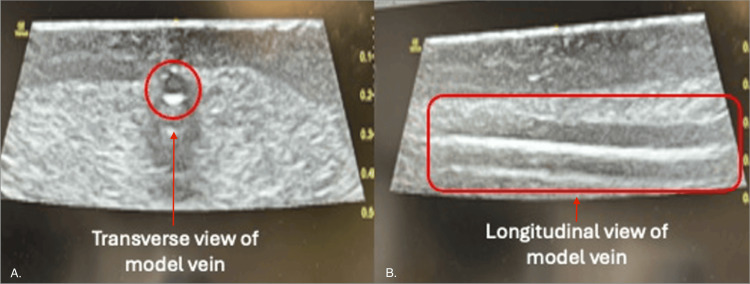
Ultrasound imaging of simulated vessels in short-axis and long-axis views using high-frequency linear probe Panel A (left image, transverse/short-axis view): key sonographic features include delineated circular anechoic lumen representing the patent vessel cavity (indicated by circular annotation), distinct vessel wall echogenicity differentiating the tubing from surrounding tissue-mimicking silicone, homogeneous echogenic background matrix (created by the 3% talcum powder additive), absence of significant posterior acoustic shadowing, adequate tissue depth visualization for the assessment of needle trajectory during simulated cannulation, and appropriate acoustic impedance matching between phantom materials enabling realistic sound wave propagation. Panel B (right image, longitudinal/long-axis view): key sonographic features include clearly visualized tubular anechoic vessel lumen extending along the scan plane (outlined by rectangular annotation), distinct parallel hyperechoic vessel walls, adequate vessel length allowing for the assessment of needle tip position along the entire vessel, consistent vessel caliber throughout the imaged segment, uniform surrounding tissue echogenicity providing realistic acoustic contrast, and appropriate imaging depth. Imaging parameters: ultrasound frequency, 10-22 MHz linear probe; imaging mode, B-mode (2D grayscale); depth setting, optimized for superficial structure visualization (typically 1-2 cm); gain, adjusted for optimal tissue-vessel contrast

Ultrasound imaging was obtained using a 10-22 MHz high-frequency linear array transducer in both cross-sectional (transverse/short-axis) and longitudinal (long-axis) orientations (Figure 4). In both views, there are distinct qualities the phantom illustrated: (1) clear patency of vessel lumens, indicated by a circular anechoic lumen in the transverse view and hyperechoic anterior and posterior vessel walls in the longitudinal view; (2) distinct echogenicity of the vessel walls which differentiates the tubing from surrounding tissue-mimicking silicone, accurately simulating the acoustic properties of neonatal soft tissue; (3) absence of significant posterior acoustic shadowing and appropriate acoustic impedance matching between phantom material, enabling realistic sound wave propagation and allowing the visualization of structures deep to the target vessel; and (4) adequate tissue depth visualization for the assessment of needle trajectory during simulated cannulation. The short-axis view permits the assessment of vessel compressibility under probe pressure, a critical skill for distinguishing venous from arterial structures. The long-axis view is critical for teaching real-time needle tip visualization during ultrasound-guided vascular access procedures.

Casting process:* *Approximately 100 mL of the final silicone mixture was prepared for each phantom. The mixture was poured gently into the mold over the embedded vessel layer, ensuring uniform fill without displacement of tubing. Curing time at room temperature was approximately 45-60 minutes. After curing, the detachable sidewalls were removed, and the phantom was inspected for defects.

Final phantom characteristics

Final iterations simulated realistic soft tissue recoil and needle resistance. Vessels were clearly visible on ultrasound in both short-axis and long-axis views using a 10-22 MHz linear probe. Phantom allowed multiple punctures without material breakdown or loss of echogenicity. It maintained shape, consistency, and performance over three months in dry storage at ambient temperature. 

This layered construction approach enabled the creation of a high-fidelity neonatal training phantom suitable for simulation-based skill acquisition, procedural competency assessment, and future educational research.

## Discussion

The choice of phantom materials was a pivotal aspect of this project, as it directly influenced the tactile realism, ultrasound compatibility, and usability of the final product. Among the materials considered, silicone polymers emerged as the most favorable option due to their durability, customizable properties, and potential for repeated needle insertions. Unlike hydrogels or gelatin-based models, silicone allowed for the precise tuning of both tactile characteristics and sonographic behavior [7]. The literature supports the use of silicone for simulation phantoms, particularly when modified with echogenic agents such as talcum powder or graphite. In our phantom, a 3% talcum powder additive provided an optimal compromise enhancing ultrasound visibility without introducing excessive acoustic shadowing or reducing pliability [8].

Hydrogels, while attractive due to affordability and ease of preparation, were deemed unsuitable for our purpose. These materials are highly perishable, degrade rapidly, and lack the mechanical integrity to support embedded vessel tubing. Their susceptibility to microbial growth further limits their practical use in educational settings that require durability and repeated use. Rubber-based models offer excellent longevity but are difficult to mold and do not replicate the soft, compliant feel of neonatal tissue. Meat-based models, although anatomically realistic, lack consistency between specimens, raise ethical concerns, and pose logistical challenges related to storage and sanitation [9-11].

By contrast, silicone offered a reproducible and modifiable platform. The incorporation of Slacker® as a silicone softener allowed us to mimic the compressibility of neonatal skin and subcutaneous tissue. However, the use of Slacker® alone reduced echogenicity, which was corrected through the addition of talcum powder. The resulting mixture of Dragon Skin™ silicone in an equal A:B ratio with 1.3 parts Slacker® and 3% talcum powder struck a balance between sonographic clarity and tactile fidelity. This formulation allowed repeated cannulation attempts without significant material degradation, and feedback from users indicated high satisfaction with both needle resistance and ultrasound image quality.

The goal of this study was to develop an anatomically appropriate and affordable ultrasound-compatible training phantom for neonatal IV access. Through careful iterative design, we created a 3D silicone phantom that replicates both the tactile and imaging characteristics of neonatal skin and vasculature. The significance of this work lies not only in the fidelity of the final product but also in its accessibility to NICUs and training centers that may not have the resources for commercial phantoms.

The development process demonstrated how critical mold architecture is in achieving consistent vessel placement and depth. Initial designs with simple holes failed to secure synthetic vessels during curing, resulting in variable geometry and poor anatomical fidelity. By integrating reinforced tubing guides into the mold's sidewalls, we were able to precisely control the positioning and tension of the embedded vessels, ensuring reproducibility across multiple casts. This attention to structural design is consistent with prior findings that emphasize the role of mold geometry in phantom realism and durability.

Material composition was another critical variable. Silicone polymers such as Dragon Skin™ are widely used in simulation because of their elasticity, biocompatibility, and longevity. However, pure silicone lacks echogenicity under ultrasound, appearing almost completely anechoic. This necessitated the addition of talcum powder, which has been shown to increase backscatter and improve the visibility of structures under ultrasound. Our findings confirm that a 3% by volume addition of talc achieves optimal echogenicity without causing internal shadowing or increased stiffness. Importantly, this mixture retained its pliability and needle-sealing properties for repeated use, making it well suited to training environments.

The addition of Slacker® served a dual purpose. First, it reduced the hardness of the silicone mixture to more closely match neonatal soft tissue. Second, it introduced a degree of "give" that mimicked the subtle resistance encountered during actual IV insertion. However, excessive Slacker® alone resulted in a loss of needle echogenicity, requiring a careful balance between tactile and imaging properties. The 1:1:1.3 Dragon Skin™ part A-to-Dragon Skin™ part B-to-Slacker® ratio emerged as the optimal compromise between usability and sonographic visibility.

The cost analysis highlights a major advantage of the developed silicone phantom: its affordability compared with commercially available neonatal vascular access trainers. The total direct cost per unit, including materials, 3D printing, and labor, was $61.70, which represents a 60-80% reduction compared with commercial simulators priced between $200 and $400. When labor is excluded, as would be the case for educational programs or simulation labs that fabricate models in-house, the material-only cost drops to just $11.70 per unit. This cost efficiency allows institutions to produce approximately 17 phantoms for the price of a single commercial trainer, making large-scale implementation feasible for residency programs, nursing education, and procedural boot camps. Importantly, the low unit cost does not compromise functional fidelity. By significantly lowering the financial barrier to accessing neonatal-specific simulation tools, this phantom has the potential to expand hands-on training opportunities and enhance procedural competency across diverse clinical training environments [12].

While our phantom was positively evaluated by clinical users, limitations remain. It does not simulate dynamic blood flow, an important feature for advanced learners. Our assessment relied on subjective feedback rather than objective performance metrics such as cannulation time or success rate. Additionally, all user testing occurred at a single institution, limiting generalizability. Future directions include integrating flow systems for flashback simulation, customizing phantoms by gestational age or anatomical variation, and validating effectiveness through multicenter studies with pre- and post-intervention assessments.

This study relied predominantly on qualitative assessment and expert user feedback rather than standardized quantitative metrics. Objective measures such as durometer hardness testing, needle insertion force quantification, and ultrasound signal-to-noise ratio analysis were not performed due to resource and equipment constraints, limiting direct comparison against commercial phantoms. However, a follow-up study examining inter-performer variability and skill acquisition metrics in a structured training environment has been conducted, providing the quantitative validation of the phantom's utility as an educational tool. The current study should therefore be interpreted as a foundational development report, with more rigorous validation forthcoming.

This study successfully developed and validated a novel 3D-printed silicone phantom system for neonatal vascular access training that achieves high fidelity across tactile, visual, and ultrasound domains while maintaining substantial cost advantages over commercial alternatives. The optimized phantom demonstrates anatomical accuracy, realistic tissue biomechanics matching published neonatal characteristics, and diagnostic-quality ultrasound imaging suitable for teaching procedural skills to both novice and experienced clinicians.

## Conclusions

The phantom addresses critical gaps in current neonatal simulation resources by providing an accessible, reproducible, and durable platform for ultrasound-guided vascular access education. High-fidelity ratings from expert clinicians, significant confidence gains among novice learners, and favorable durability characteristics support its potential for widespread implementation in medical education programs. By documenting detailed fabrication protocols and openly sharing methodologies, this work enables replication and adaptation by institutions seeking cost-effective solutions for procedural training.
